# The Effect of Progressive Dynamic Balance Training on Physical Function, The Ability to Balance and Quality of Life Among Elderly Women Who Underwent a Total Knee Arthroplasty: A Double-Blind Randomized Control Trial

**DOI:** 10.3390/ijerph18052513

**Published:** 2021-03-03

**Authors:** Heon-Gyu Lee, Jungae An, Byoung-Hee Lee

**Affiliations:** 1 Graduate School of Physical Therapy, Sahmyook University, Seoul 01795, Korea; slehfldlrk@naver.com; 2 Department of Physical Therapy, Seoul Now Hospital, Seongnam 13591, Korea; jungean@hotmail.com; 3 Department of Physical Therapy, Sahmyook University, Seoul 01795, Korea

**Keywords:** total knee arthroplasty, dynamic balance training, physical function, quality of life

## Abstract

Total knee arthroplasty (TKA) is used to treat end-stage osteoarthritis. However, this surgical procedure affects the mechanical receptor function and impairs the ability to balance. Dynamic balance training has been reported to improve stability and self-confidence and safely yield increased physical activity. This study aimed to investigate the effect of dynamic balance training on physical function, the ability to balance and quality of life among patients who underwent TKA. Thirty-eight participants were assigned to either the progressive dynamic balance training (PDBT) with physical therapy group (*n* = 19) or the control group (*n* = 19). The experimental group undertook a dynamic balance program with physical therapy for 30 minutes per day, five times per week for six weeks, while the control group undertook physical therapy only. A continuous passive motion exercise was performed for 20 minutes after training by both groups. The outcomes were evaluated using the Western Ontario and McMaster Universities (WOMAC) Osteoarthritis Index, pain pressure threshold (PPT), range of motion (ROM), Knee Outcome Survey-Activities of Daily Living (KOS-ADLS), Multifunction Force Measuring Plate, timed up and go (TUG) test and Short-Form Health Survey 36 (SF-36). Physical function (WOMAC Osteoarthritis Index, ROM and KOS-ADLS score) and the ability to balance (TUG test score, confidence ellipse area, path length and average velocity) significantly improved (*p* < 0.05) in the experimental group compared with the control group. In contrast, the physical component summary score for the SF-36 regarding quality of life significantly improved (*p* < 0.05); however, the mental component summary score for the SF-36 and PPT did not significantly differ between the groups. Therefore, we suggest that PDBT with physical therapy has positive effects on physical function, the ability to balance and quality of life among patients who underwent TKA.

## 1. Introduction

Total knee arthroplasty (TKA) is used to treat patients with end-stage osteoarthritis who experience pain and struggle to perform activities of daily living owing to having degenerative knee joints [1,2]. Although TKA can improve the quality of life of patients, some may experience a decrease in their proprioception and the ability to balance after surgery [3] and there is a significant incidence of unsatisfactory post-operative outcomes [4]. Thirty-seven percent of patients have limited improvement in their function at first year post-surgery. Most commonly, patients report a reduction in their walking speed, difficulty going up and down the stairs and an inability to return to the same level of pre-operative sports performance [5,6,7,8]. Therefore, patients who have undergone TKA can have functional limitations with impaired motor control and ability to balance. During TKA, some tendons and capsules are tightened to restore the joint space that has deteriorated owing to osteoarthritis. Additionally, a few ligaments are removed to restore the intracapsular structures. These changes affect the mechanical receptor function and impair balance and motor control [9].

The ability to balance is the main factor that enables individuals to maintain posture and react to perturbations. Dynamic balance refers to balance control with movement and is an important aspect of overall balance functions [10]. The ability to balance among patients with knee pain who underwent surgery has been reported to be significantly weaker than that among those who had not undergone knee surgery. These patients had lower balance and mobility test scores and a higher risk of falls [11,12,13]. Balance and muscle strength tests can be used to assess and monitor an individual’s health over time and predict multi-morbidity, dependence in performing basic activities of daily living (ADLs) and early mortality. Both tests are also of a substantial value in predicting the future health status and functional performance in older adults [14,15].

Previous training for patients who have undergone TKA have been demanded to complete exercises that include sitting on a chair, squatting, climbing the stairs, and walking but these activities do not provide full support to consequently improve the functional ability and motor control of TKA patients [16]. It is then necessary to undertake dynamic balance training, which reflects movements and activities that occur in daily life such as twisting, turning, stopping, standing on unstable surfaces, changing speed and changing direction to improve these functions [17]. Dynamic balance training improves stability and self-confidence, safely yields increased physical activity and ultimately results in long-term benefits among patients who have undergone TKA [16,17,18]. Traditionally, quadriceps strength, flexibility, balance training has been used for TKA rehabilitation. Johnson et al. reported that whole body vibration(WBV)exercise performed for three to six weeks after TKA improved knee extensor strength and the timed up and go (TUG) test scores and that the effect was related to less stress induced during unweighted static and dynamic exercise on a vibratory platform [19]. However, the evidence regarding the full effect of dynamic balance training with physical therapy in patients who have undergone TKA is limited. Thus, this study aimed to investigate the effect of six weeks of progressive dynamic balance training (PDBT) on physical function, the ability to balance and quality of life among patients who underwent TKA.

## 2. Materials and Methods

### 2.1. Study Design

This double-blind, randomized control trial was conducted to determine the effect of a six weeks progressive dynamic balanced training program on physical function, the ability to balance and quality of life among patients who underwent TKA at a local orthopedic surgery and rehabilitation hospital.

### 2.2. Subjects

**Figure 1:**
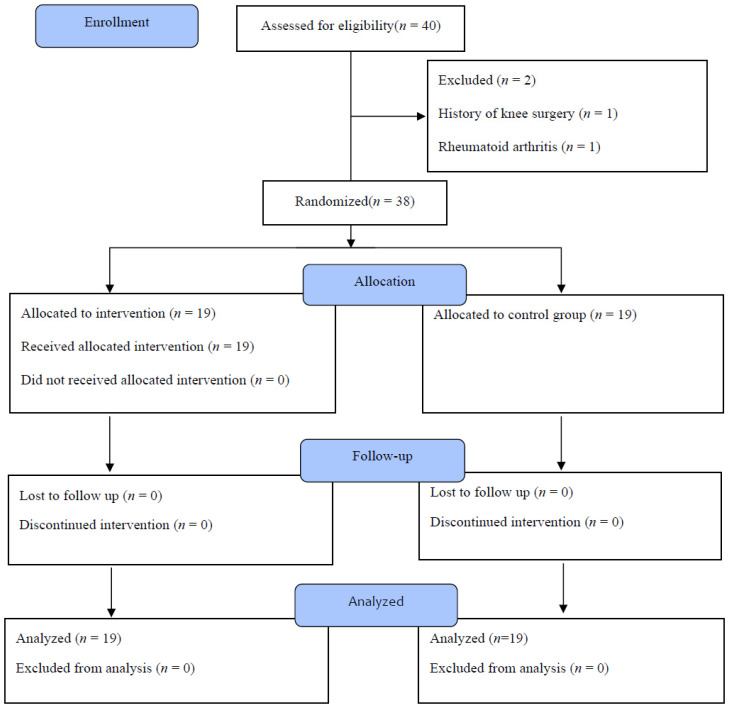
Consort flowchart diagram of the study participants.

Thirty-eight female patients were randomly allocated to the PDBT group (*n* = 19) and the control group (*n* = 19). The inclusion criteria for this study were a history of TKA and ability to walk more than 10 meters and understand instructions given during the experimental procedure. Conversely, the exclusion criteria were age over 80 years, a history of knee surgery within six months, rheumatoid arthritis or cognitive deficiency. All participants underwent a minimally invasive quadriceps-sparing tricompartmental, cemented TKA using a high-flexion mobile prosthesis. One senior surgeon conducted all of the procedures (Figure 1).

### 2.3. Ethical Statement

This study was conducted in accordance with the Declaration of Helsinki, approved by the institutional review board of Sahmyook University in the Republic of Korea (2-7001793-N-012018057HR) and retrospectively registered at the Clinical Research Information Service in the Republic of Korea (KCT00005129). All experimental protocols and procedures were explained to each subject and written informed consent was obtained before enrollment in the study.

### 2.4. Experimental Procedures

The participants were selected in accordance with the inclusion and exclusion criteria. All of the participants provided their informed consent in relation to the exercise method and schedule for this study. Pre-tests were performed a week before the program started.

The experimental group undertook a dynamic balance training program (PDBT) with physical therapy for 30 minutes per day, five times per week for six weeks. The control group only undertook general physical therapy. Training began on the third day after surgery and the exercise intensity was gradually increased over six weeks. The participants were supported by a knee protector and a walker. The six weeks progressive balance exercise program consisted of four exercise levels. In the first and second weeks, the participants twisted their torso while sitting on a chair and standing up and lifted their heels while standing. In the third and fourth weeks, they twisted their torso while standing, walked sideways and used a step box to step up and down. In the fifth week, they marched, marched with turns, used a step box to step sideways and practiced tandem gait. In the sixth week, they walked around a cone, practiced tiptoe gait and changed directions while walking. The total exercise time was 30 minutes each session with an inter-rest time of 30–40 seconds and two sets of 10 for each movement [16,20]. Additionally, all patients underwent an inpatient rehabilitation program after TKA.

### 2.5. Outcome Measurements

Physical function was evaluated using the Western Ontario and McMaster Universities (WOMAC) Osteoarthritis Index, pain pressure threshold (PPT), range of motion (ROM) and Knee Outcome Survey-Activities of Daily Living (KOS-ADLS). The WOMAC Osteoarthritis Index is a reliable tool for evaluating patients with knee and hip osteoarthritis who have undergone TKA (ICC = 0.92) and specifically pain, stiffness and physical function. It has a total score of 96 points with lower scores indicating fewer symptoms and less physical disability [21]. The KOS-ADLS is a self-reporting questionnaire (ICC = 0.97) that evaluates knee symptoms and functional limitations on ADLs in a total of 14 categories.

Balance was evaluated via static and dynamic balance assessments. The static ability to balance was evaluated using the PDM Multifunction Force Measuring Plate (Zebris Medical, Germany, 2016) and the dynamic ability to balance used the TUG test. The PDM Multifunction Force Measuring Plate (ICC = 0.83–0.99) is a flat force plate with one force sensor per cm² that each independently measures the static and dynamic pressures of the foot while standing. The measuring pressure ranges from 1 to 120 N/cm² with a static sampling pressure extraction speed of 2–5 Hz, a dynamic sampling pressure extraction speed of approximately 90 Hz and an accuracy of ± 5%. The measurements were taken three times and the mean values were calculated using the confidence ellipse area (CEA), path length (PL) and average velocity (AV) [22]. The TUG test was performed with the patients sitting in a chair without armrests that was 50 cm in height. In this test, the participants stood up at the same time as the tester’s signal, proceeded to a 3 m point in front of the chair and then sat back down [23]. The average value of three repetitions was obtained.

Quality of life was assessed using the Short-Form Health Survey 36 (SF-36) divided into physical and mental sections (ICC = 0.94). Among the 36 questions, the physical component consists of 10 items on physical function, four on physical role and two on physical pain (16 questions). The mental component is divided into three items on emotional role and six items on mental health. The other five health questions include four items on vitality and two on social function that contribute to physical and mental health. The higher the score, the higher the quality of life [24,25,26].

### 2.6. Sample Size and Statistical Analysis

G*Power version 3.1.9.7 (Heinrich-Heine-Universität, Düsseldorf, Germany) was used to perform a power analysis for estimating the sample size; the overall effect size index for all of the outcome measures and power of the study were 0.50, a probability of 0.05 and to minimize a type II error (power of 80%). As the estimated target sample size was 34 with a considered dropout rate of 25%, 40 participants were recruited for this experiment.

The Statistical Package for the Social Sciences version 19 (IBM, Chicago, IL, USA) was used to perform all of the statistical analyses. Results are presented as means ± standard deviations. The Shapiro–Wilk test was used to analyze the clinical and general characteristics of the subjects to assess the normality of data distribution. An independent *t*-test was performed to determine the average difference between the two groups. A paired *t*-test was used to compare the difference between the pre- and post-intervention results within each group. For all tests, the statistical significance was set at *p* values of 0.05.

## 3. Results

Table 1 shows the general characteristics and homogeneity test results of the study subjects. For the baseline physical function, balance and quality of life scores, no significant differences were found between the groups (*p* < 0.05).

### 3.1. Physical Function and Pain

The WOMAC Osteoarthritis Index for pain, stiffness and physical function improved in the PDBT group (*p* < 0.001); there was a significant difference found in all items of the WOMAC questionnaire between the groups (*p* < 0.01). The ROM increased from 107.56° to 138.87° in the PDBT group and from 115.32° to 136.96° in the control group (*p* < 0.001); there was a significant difference found between the groups (*p* < 0.01). In the comparison between the pre- and post-intervention KOS-ADLS scores, there was a significant difference found in both groups (*p* < 0.001); there was also a significant difference between the groups (*p* < 0.05). In contrast, in the comparison between the pre- and post-intervention PPTs, a significant difference was found (*p* < 0.01); however, there was no significant difference noted between the groups. Table 2 describes the outcomes related to physical function and pain.

### 3.2. Static and Dynamic Balance

The TUG test was performed and the force plate was used to measure the ability to balance. Changes in the ability to balance are shown in Table 3. For the static ability to balance, the CEA, PL and AV significantly improved after the intervention in the PDBT group (*p* < 0.01); there were significant differences in the improvements in these parameters between the groups (*p* < 0.05). For the dynamic ability to balance, the parameters significantly improved after the intervention in both groups (*p* < 0.001); the PDBT group showed greater significant improvements than did the control group (*p* < 0.05).

### 3.3. Quality of Life

Table 4 shows the overall quality of life of the participants. The physical component summary scores significantly improved after the intervention in the PDBT group (*p* < 0.001) and significant differences were noted between the groups (*p* < 0.01).

## 4. Discussion

### 4.1. Physical Function and Pain

The physical function of patients who have undergone TKA is an important factor not only for ensuring the sagittal movement of the knee but also for performing daily living tasks such as climbing up and down the stairs, standing from a seated position and squatting [27]. Although TKA decreases pain and improves quality of life, some patients still complain of not being able to complete ADLs after surgery [28].

During TKA, the knee ligaments and cartilage are removed to restore the joint space exacerbated by arthritis, which is believed to affect the function of mechanoreceptors and consequently impair motor control [29,30]. An improvement in the ability to balance so as to recover functional impairment after TKA contributes to mechanical receptor stimulation [31]. Therefore, dynamic balance exercises should be considered to help improve these dysfunctions. This study conducted PDBT for six weeks and found improvements in the physical function parameters. A few previous studies have conducted balance training in patients who underwent TKA and found significant differences in the physical function scores such as the WOMAC Osteoarthritis Index, TUG test score and KOS-ADLS score and findings for one-leg standing, climbing up and down stairs and 10 m gait [32,33]. They suggested that balance training could help reduce the functional limitations for these patients. This effect may be associated with the muscle being activated during dynamic balance training, which helps maintain postural ability by increasing the functional status or static muscular contraction force of the knee in the early phase after TKA [34]. In addition, repetitive balance training related to ADLs including climbing up and down the stairs and practicing tandem and tiptoe gaits affects the muscle activity of the lower limbs, which leads to improvements in the KOS-ADLS score. Although a significant difference was noted in physical function, no significant difference was noted in the pressure pain between the groups. Skou et al. [35] evaluated 54 patients who had undergone TKA and observed a significant improvement from 15.6 kg/cm^2^ before surgery to 6.69 kg/cm^2^ after surgery. Similarly, in previous studies, dynamic balance training improved knee pain and a significant difference was found between groups [16,20,36,37]. These results could be attributed to dynamic balance training being applied to patients in the early phase after surgery and nonsteroidal anti-inflammatory drugs and local anesthetics being used to control pain [38,39]; conversely, previous studies have commenced training between three months and one year after surgery [17].

### 4.2. Balance

In this study, we used a force plate to objectively evaluate the static ability to balance and performed the TUG test to evaluate the dynamic ability to balance; consequently, we found significant improvements in the parameters. The experience of repetitive training affecting motor response and the stimulation of proper proprioception and visual cues significantly improved the static and dynamic abilities to balance; furthermore, balance training conducted among elderly patients at risk of falls improved their ability to walk, balance and perform activities [40,41]. Gusi et al. [42] observed a significant difference in the ability to balance and knee muscle strength in elderly patients after balance training. Piva et al. [16] also noted a significant difference in the abilities to balance and walk after applying a balance exercise program to patients who had undergone TKA. Directional walking, which requires the ability to dynamically balance, is an essential activity in daily living; it also requires more sensorimotor skills because it is necessary to distribute weight equally inside and outside both feet [43]. Therefore, patients who have undergone TKA need continuous dynamic balance training to maintain their balance and prevent falls after they are discharged.

### 4.3. Quality of Life

This study observed a significant difference in the improvement in the overall score of quality of life between the groups. However, a significant difference was noted only in the physical component summary scores. The dynamic balance training program combined with physical therapy contributed to the recovery of physical functions and the ability to balance, enabling patients to undertake basic cleaning of their house and perform light exercise and gait-related movements. Moffet et al. [44] identified significant differences in the improvement in quality of life after applying functional training to patients who had undergone TKA after two months. Petterson et al. [45] observed an improvement in quality of life after applying physical therapy for six weeks to patients four weeks after TKA. They found that difficulties related to completing physical ADLs were reduced. However, promoting physical activity in patients with mental illness can alleviate symptoms such as low self-esteem and depression [46]. Although an increasing number of studies have been conducted that focus on the effects of physical activity on mental health, its exact effects have not yet been confirmed [47]. Physical activity and dynamic exercise may play an important role in moderate depression or mild anxiety; however, studies conducted on this topic in the elderly population are limited [48]. Additionally, previous studies have measured quality of life several months after surgery while our study measured such at six weeks after surgery. Patients who underwent TKA during this period did not show significant improvements in depression and anxiety attributed to post-operative pain [49]. Therefore, during this initial period, dynamic balance training could help improve patients’ physical functions, ROM and, consequently, the physical aspect of quality of life. However, in this early stage, depression and anxiety are not improved owing to pain after TKA. It is then necessary to develop a physical activity training program that improves the overall quality of life by determining a method to improve depression and anxiety along with the patients’ physical abilities.

This study had several limitations. The number of included patients who had undergone TKA was relatively small and all subjects were women and had undergone bilateral TKA, which does not represent a typical sample of patients who have undergone TKA. Additionally, the patients were not followed up over a long period, considering that the study period was only six weeks. Therefore, it is difficult to confirm whether the effect was maintained in the long term. Patients’ diet and amount of medication taken were not controlled as the evaluation period was limited to the early phase after TKA. Therefore, further studies with a larger number of subjects and a more extended follow-up period are required to better ascertain the effects of dynamic balance training combined with physical therapy in patients who have undergone TKA.

## 5. Conclusions

This study showed that PDBT combined with physical therapy improved the physical function, the ability to balance and quality of life among patients who underwent TKA. Based on this evidence, we suggest that PDBT is an effective intervention for preventing falls and improving the ability to perform ADLs and quality of life after TKA.

## Figures and Tables

**Table 1:** Demographic data of the two groups (*n* = 38).

	PDBT Group	Control Group	*χ^2^/t (p)*
	(*n* = 19)	(*n* = 19)	
Age (year)	72.05 (5.15)	71.89 (5.44)	0.092 (0.927)
Height (cm)	150.95 (5.97)	151.97 (5.86)	0.532 (0.598)
Weight (kg)	57.60 (7.39)	61.539 (12.45)	1.183 (0.246)
BMI (kg/m^2^)	25.27 (2.67)	26.75 (4.03)	1.333 (0.191)

Values are presented as means (standard deviations). PDBT: progressive dynamic balance training; BMI: body mass index.

**Table 2:** Comparison of physical function within and between the groups (*n* = 38).

Parameters		PDBT Group	Control Group (*n* = 19)	t (*p*)
		(*n* = 19)		
WOMAC Osteoarthritis Index (Total)	Baseline	64.00 (7.431)	58.26 (14.854)	1.506 (0.141)
	Post-intervention	4.05 (1.77)	7.16 (2.67)	
	Baseline/post-intervention (Difference)	6.95 (2.09)	3.89 (4.04)	2.924 (0.007) *
	t (*p*)	14.460 (0.000) **	4.202 (0.001) *	
WOMAC Osteoarthritis Index (Pain)	Baseline	11.00 (2.49)	11.05 (3.02)	0.058 (0.954)
	Post-intervention	4.05 (1.77)	7.16 (2.67)	
	Baseline/post-intervention (Difference)	6.95 (2.09)	3.89 (4.04)	2.924 (0.007) *
	t (*p*)	14.460 (0.000) **	4.202 (0.001) *	
WOMAC Osteoarthritis Index (Stiffness)	Baseline	4.74 (1.32)	4.00 (1.41)	1.656 (0.106)
	Post-intervention	1.53 (0.77)	2.00 (0.81)	
	Baseline/post-intervention (Difference)	3.21 (1.31)	2.00 (1.10)	3.070 (0.004) *
	t (*p*)	10.637 (0.000) **	7.886 (0.000) **	
WOMAC Osteoarthritis Index (Physical Function)	Baseline	48.26 (6.37)	43.21 (11.50)	1.674 (0.103)
	Post-intervention	23.53 (4.46)	28.11 (8.67)	
	Baseline/post-intervention (Difference)	24.74 (6.00)	15.11 (12.68)	2.991 (0.006) *
	t (*p*)	17.948 (0.000) **	5.191 (0.000) **	
ROM (°) Knee Flexion	Baseline	107.56 (9.92)	115.32 (14.71)	1.907 (0.064)
	Post-intervention	138.87 (5.99)	136.96 (7.99)	
	Baseline/post-intervention (Difference)	31.31 (9.52)	21.63 (12.98)	2.619 (0.013) *
	t (*p*)	14.335 (0.000) **	7.266 (0.000) **	
PPT (kg/cm^2^)	Baseline	2.80 (0.76)	2.40 (0.67)	1.714 (0.095)
	Post-intervention	3.60 (0.91)	2.96 (0.94)	
	Baseline/post-intervention (Difference)	0.79 (1.15)	0.56 (0.93)	0.685 (0.497)
	t (*p*)	3.002 (0.008) *	2.631 (0.017) *	
KOS-ADLS score	Baseline	26.05 (11.51)	28.84 (9.74)	0.806 (0.425)
	Post-intervention	46.47 (8.68)	42.79 (7.77)	
	Baseline/post-intervention (Difference)	20.42 (8.94)	13.95 (8.34)	2.308 (0.027) *
	t (*p*)	9.957 (0.000) **	7.287 (0.000) **	

Values are presented as means (standard deviations). PDBT: progressive dynamic balance training; WOMAC: Western Ontario and McMaster Universities; ROM: range of motion; KOS-ADLS: Knee Outcome Survey-Activities of Daily Living; PPT: pain pressure threshold. * Statistically significant (*p* < 0.05). ** Statistically significant (*p* < 0.001).

**Table 3:** Comparison of the ability to balance within and between the groups (*n* = 38).

		PDBT Group	Control Group	t (*p*)
		(*n* = 19)	(*n* = 19)	
CEA (mm^2^)	Baseline	475.28 (244.36)	448.80 (232.29)	0.342 (0.734)
	Post-intervention	269.62 (172.90)	351.94 (174.83)	
	Baseline/post-intervention (Difference)	205.65 (193.42)	96.85 (127.38)	2.048 (0.048) *
	t (*p*)	4.635 (0.000) **	3.314 (0.004) *	
PL (mm)	Baseline	133.58 (49.49)	125.20 (42.85)	0.558 (0.580)
	Post-intervention	104.50 (48.12)	127.27 (49.48)	
	Baseline/post-intervention (Difference)	29.08 (41.66)	2.07 (37.67)	2.417 (0.021)^*^
	t (*p*)	3.042 (0.007) *	0.240 (0.813)	
AV (mm/s)	Baseline	14.20 (5.10)	12.83 (5.00)	0.835 (0.409)
	Post-intervention	10.78 (4.57)	12.34 (7.08)	
	Baseline/post-intervention (Difference)	3.42 (3.58)	0.49 (3.49)	2.552 (0.015) *
	t (*p*)	4.166 (0.001) *	0.612 (0.548)	
TUG (s)	Baseline	13.58 (2.72)	13.02 (2.15)	0.700 (0.489)
	Post-intervention	9.49 (0.99)	10.93 (1.47)	
	Baseline/post-intervention (Difference)	4.08 (2.42)	2.09 (1.93)	2.799 (0.008) *
	t (*p*)	7.357 (0.000) **	4.720 (0.000) **	

Values are presented as means (standard deviations). PDBT: progressive dynamic balance training; CEA: confidence ellipse area; PL: path length; AV: average velocity; TUG: timed up and go. * Statistically significant (*p* < 0.05). ** Statistically significant (*p* < 0.001).

**Table 4:** Comparison of quality of life within and between the groups (*n* = 38).

		PDBT Group	Control Group	t (*p*)
		(*n* = 19)	(*n* = 19)	
SF-36	Baseline	75.71 (20.47)	83.87 (20.74)	1.220 (0.230)
Total Score	Post-intervention	124.92 (20.63)	106.74 (15.85)	
	Baseline/post-intervention (Difference)	49.21 (22.36)	22.87 (26.83)	3.286 (0.002) *
	t (*p*)	9.593 (0.000) **	3.715 (0.002) *	
SF-36	Baseline	32.40 (11.67)	37.03 (10.45)	1.286 (0.207)
PCS	Post-intervention	64.47 (10.73)	52.23 (8.83)	
	Baseline/post-intervention (Difference)	32.06 (12.68)	15.20 (11.33)	4.319 (0.000) **
	t (*p*)	11.017 (0.000) **	5.844 (0.000) **	
SF-36	Baseline	43.30 (12.14)	46.83 (13.71)	0.841 (0.406)
MCS	Post-intervention	60.45 (12.51)	54.50 (9.33)	
	Baseline/post-intervention (Difference)	17.15 (11.98)	7.66 (19.62)	1.797 (0.081)
	t (*p*)	6.238 (0.000) **	1.703 (0.106)	
SF-36	Baseline	75.71 (20.47)	83.87 (20.74)	1.220 (0.230)
Total Score	Post-intervention	124.92 (20.63)	106.74 (15.85)	
	Baseline/post-intervention (Difference)	49.21 (22.36)	22.87 (26.83)	3.286 (0.002) *
	t (*p)*	9.593 (0.000) **	3.715 (0.002) *	

Values are presented as means (standard deviations). SF-36: Short-Form Health Survey 36; PCS: physical component summary; MCS: mental component summary. ^*^ Statistically significant (*p* < 0.05). ^**^ Statistically significant (*p* < 0.001).

## Data Availability

Not applicable.
